# Using tree diversity to compare phylogenetic heuristics

**DOI:** 10.1186/1471-2105-10-S4-S3

**Published:** 2009-04-29

**Authors:** Seung-Jin Sul, Suzanne Matthews, Tiffani L Williams

**Affiliations:** 1 Department of Computer Science and Engineering, Texas A&M University, College Station, Texas, USA

## Abstract

**Background:**

Evolutionary trees are family trees that represent the relationships between a group of organisms. Phylogenetic heuristics are used to search stochastically for the best-scoring trees in tree space. Given that better tree scores are believed to be better approximations of the true phylogeny, traditional evaluation techniques have used tree scores to determine the heuristics that find the best scores in the fastest time. We develop new techniques to evaluate phylogenetic heuristics based on both tree scores and topologies to compare Pauprat and Rec-I-DCM3, two popular Maximum Parsimony search algorithms.

**Results:**

Our results show that although Pauprat and Rec-I-DCM3 find the trees with the same best scores, topologically these trees are quite different. Furthermore, the Rec-I-DCM3 trees cluster distinctly from the Pauprat trees. In addition to our heatmap visualizations of using parsimony scores and the Robinson-Foulds distance to compare best-scoring trees found by the two heuristics, we also develop entropy-based methods to show the diversity of the trees found. Overall, Pauprat identifies more diverse trees than Rec-I-DCM3.

**Conclusion:**

Overall, our work shows that there is value to comparing heuristics beyond the parsimony scores that they find. Pauprat is a slower heuristic than Rec-I-DCM3. However, our work shows that there is tremendous value in using Pauprat to reconstruct trees—especially since it finds identical scoring but topologically distinct trees. Hence, instead of discounting Pauprat, effort should go in improving its implementation. Ultimately, improved performance measures lead to better phylogenetic heuristics and will result in better approximations of the true evolutionary history of the organisms of interest.

## Background

Phylogenetics is concerned with inferring the genealogical relationships between a group of organisms (or taxa). These evolutionary relationships are typically depicted in a binary tree, where leaves represent the organisms of interest and edges represent the evolutionary relationships. Phylogenetic trees have been used successfully in designing more effective drugs, tracing the transmission of deadly viruses, and guiding conservation and biodiversity efforts [1,2]. The grand challenge problem in phylogenetics is reconstructing the *Tree of Life*, the evolutionary history of the 10 to 100 million estimated organisms on earth. However, inferring evolutionary trees is not a trivial task. Since the true evolutionary history for a set of organisms is unknown, the problem is often reformulated as an NP-hard optimization problem. Trees are given a score, where trees with better scores are believed to be better approximations of the true evolutionary history. For n taxa, there are an exponential number of evolutionary hypotheses: (2*n *- 3)!! possible solutions to be exact. As a result, an exhaustive exploration of the space of possible solutions (or "tree space") is infeasible. Thus, the most popular techniques in the field use heuristics to reconstruct phylogenetic trees.

In this paper, we develop new techniques to compare the performance of phylogenetic heuristics. While phylogenetic heuristics are used to search stochastically for the best trees in tree space, their results often vary across each run of the heuristic. As a result, it is difficult to compare performance among heuristics that produce different solutions. Our work evaluates phylogenetic heuristics based on both the *score *and *topology *of the trees found. More specifically, our work centers around the following two questions.

1. What value (if any) do slower heuristics provide?

2. How effective are parsimony scores in distinguishing between different tree topologies?

Traditional techniques for comparing phylogenetic heuristics use convergence plots to show how the best score improves over time, as best scores are thought to symbolize more accurate trees. Under this measure, the heuristic that obtains the best score in the fastest time is desired. Given that different tree topologies may have identical tree scores, preference of good-scoring trees found by fast heuristics may result in overlooking potentially more accurate evolutionary histories that were found by slower approaches.

We consider the performance of two well-known Maximum Parsimony (MP) search heuristics, Parsimony Ratchet [3] and Recursive-Iterative DCM3 (Rec-I-DCM3) [4] on four molecular datasets of 60, 174, 500 and 567 taxa. The parsimony ratchet algorithm used in this paper is called Pauprat since we used a Perl script by Bininda-Emonds [5] to generate a PAUP* [6] batch file to run the parsimony ratchet heuristic. Our first observation is that there are benefits to considering different speed heuristic implementations of a MP phylogenetic analysis. In general, Pauprat is a slower heuristic than Rec-I-DCM3. Since we were curious of the merits of a heuristic, time constraints were removed from consideration in this study. However, both Pauprat and Rec-I-DCM3 find different trees with the same best parsimony scores. These diverse best-scoring trees denote that the heuristics are visiting different areas of the exponentially-sized tree space. We note that although TNT [7] has a faster implementation of parsimony ratchet than PAUP*, TNT does not have the capability to return to the user the set of trees found during each iterative step of the parsimony ratchet algorithm. The Pauprat implementation of parsimony ratchet provides this capability. Moreover, the Rec-I-DCM3 implementation also provides users with the trees found during each step of the algorithm.

Secondly, although different trees are found with the same parsimony score, it's interesting to consider whether maximum parsimony is effectively distinguishing between the trees, which has significant implications for understanding evolution. By using a measure called *relative entropy*, we show that for a given collection of trees, parsimony scores have less information content than topological distance measures such as Robinson-Foulds (RF) distance [8]. In other words, for a collection of trees, parsimony scores identify fewer unique trees—which increases the potential of being stuck in a local optimum and producing less accurate phylogenies—than topological distance measures. Thus, more powerful search strategies could be designed by using a combination of score and topological distance to guide the search into fruitful areas of the exponentially-sized tree space.

## Results and discussion

### Frequency of the top-scoring trees

Table 1 shows the number of trees found by the Pauprat and Rec-I-DCM3 heuristics in terms of the number of steps they are from the best score, *b*, we found across the algorithms. For each dataset, both Pauprat and Rec-I-DCM3 find trees with the best score, *b*. Let *x *represent the parsimony score of a tree *T *. Then, tree *T *is *x *- *b *steps away from the best score. In Table 1, step_0_, step_1_, and step_2 _represent trees that are 0, 1 and 2 steps away from the best score, *b*, respectively. It is clear that the top-scoring trees from Pauprat comprise a large proportion of the total collection of its 5,000 trees for the smaller datasets (60 and 174 taxa). On the other hand, the top trees for Rec-I-DCM3 comprise the majority of its collection of trees for the larger datasets (500 and 567 taxa).

**Table 1:** Frequency of the top-scoring trees from Pauprat and Rec-I-DCM3.

		Pauprat				Rec-I-DCM3			
		
No. of taxa	best score	step_0_	step_1_	step_2_	% of total	step_0_	step_1_	step_2_	% of total
60	8,698	1,508	0	1,509	60.3%	59	0	343	8.0%
174	7,440	2,626	1,042	635	86.1%	170	491	1,301	39.2%
500	16,218	184	562	955	34.0%	1,231	1,279	983	69.9%
567	44,165	27	263	735	22.5%	1,299	1,671	903	77.5%

The table shows the number of top-scoring trees found by each algorithm. step_*i *_refers to trees that are *i *steps away from the best score. Hence, step_0 _represents the number best-scoring trees, step_1 _are trees one step away from the best, and step_2 _are trees with parsimony scores two steps greater than the best score. Each algorithm found 5,000 total trees. For Pauprat (Rec-I-DCM3), the step_0_, step_1_, and step_2 _trees make up 67.6% (10.7%) of the 5,000 total trees in the collection for the 60 taxa dataset. For the 567 taxa dataset, the top-scoring trees represent 22.5% and 77.5% of the 5,000 trees found by Pauprat and Rec-I-DCM3, respectively.

### Topological comparisons of top-scoring trees

**Figure 1:**
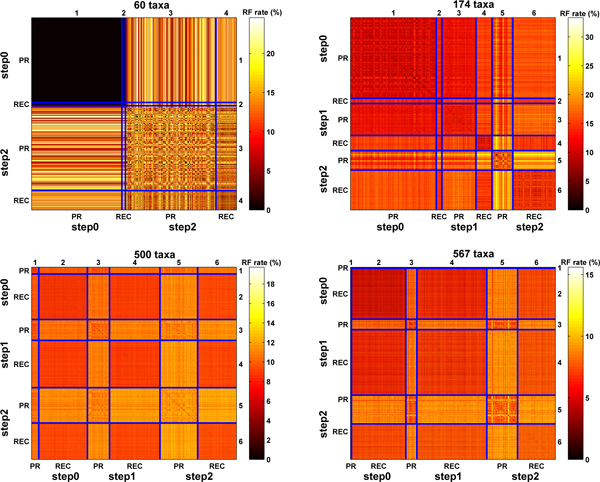
**Comparing the RF rates of the top-scoring trees found by Pauprat and Rec-I-DCM3**. Each heatmap reflects the RF matrix between all step0, step1, and step2 trees found by Pauprat and Rec-I-DCM3. The heatmaps for the 60, 174, 500, and 567 taxa datasets represent the 3419 × 3419, 6265 × 6265, 5194 × 5194, and 4898 × 4898 RF matrices, respectively. The RF rate axis varies across the heatmaps.

Figure 1 shows the topological differences (measured by the RF rate) between the top-scoring trees found by the two phylogenetic search heuristics. We use a heatmap representation of the RF matrix, where each value (cell) in the two-dimensional *t *× *t *RF matrix is represented as a color. Darker (lighter) colors represent smaller (higher) RF rates. Since the RF matrix is symmetric, our heatmap is symmetric as well. For each heatmap, the right values are *x *coordinates and the values on the top are *y *coordinates. Consider the heatmap that represents the collection of 60 taxa trees. Cell (1, 1) represents the set of step_0 _trees from the Pauprat heuristic. Each of the 1,508 step_0 _trees is compared to each other. Their resulting RF rates are 0%, which is denoted by a black coloring of the 1,508 × 1,508 block of cells. Hence, the best scoring trees found by the Pauprat heuristic are identical. A similar conclusion can be made concerning the 59 step_0 _trees found by Rec-I-DCM3 and denoted by cell (2, 2) in the heatmap. If we look at the step_0 _trees from both Pauprat and Rec-I-DCM3, represented by cells (*x*, *y*), where *x*, *y *≤ 2, the entire block of cells have a RF rate of 0%. Hence, for the 60 taxa dataset, the heuristics found identical best-scoring trees. For the step_2 _trees, reflected in cells (3, 3) and (4, 4), there is more variation among the 60 taxa trees. Neither heuristic found step_1 _trees. The heatmap also compares trees with different number of steps to the best. For example, cell (1, 4) compares step_0 _Pauprat trees with step_2 _Rec-I-DCM3 trees and denotes that they have a wider range of topological differences between them.

Although the best trees are identical in the 60 taxa dataset, the heatmaps in Figure 1 show that the Pauprat and Rec-I-DCM3 algorithms find topologically different trees that have the same parsimony score. Furthermore, as the parsimony score increases, there is more variety in the topological structure of the step_1 _and step_2 _trees. The top-scoring trees found by Rec-I-DCM3 algorithm are more similar to each other than their Pauprat counterparts. Finally, the heatmaps show that Pauprat finds more topologically dissimilar trees than Rec-I-DCM3. Thus, the Rec-I-DCM3 step_*i *_trees tend to form clusters that are distinct from the step_*i *_Pauprat trees.

**Figure 2:**
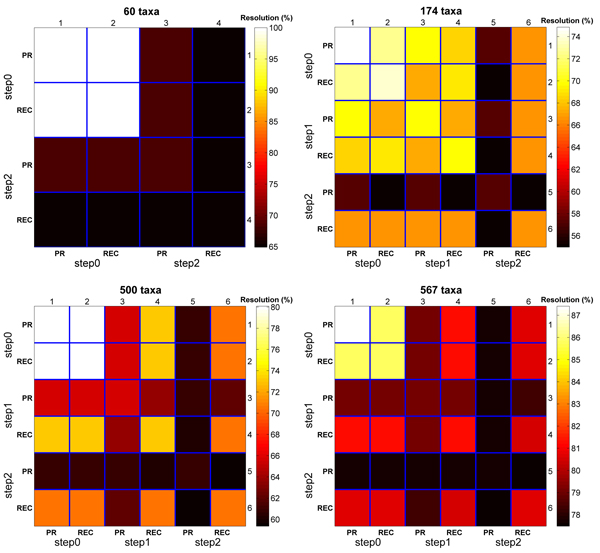
**Comparing the strict resolution rates of the top-scoring trees**. Each 6 × 6 heatmap represents the strict resolution rate between the step_0_, step_1_, and step_2 _trees found by the Pauprat and Rec-I-DCM3 heuristics.

**Figure 3:**
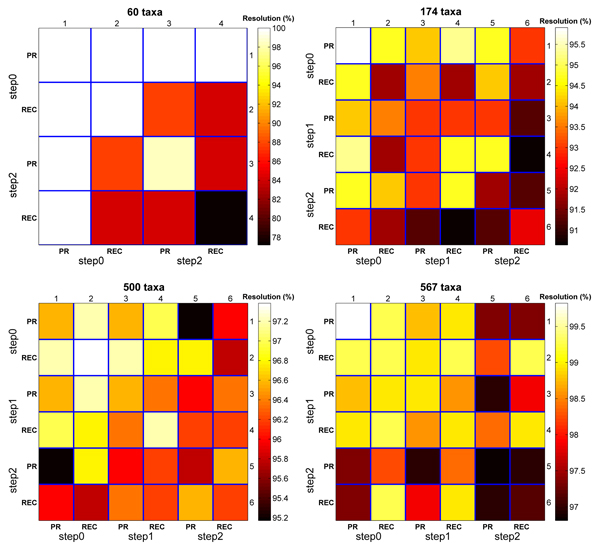
**Comparing the majority resolution rates of the top-scoring trees**. Each 6 × 6 heatmap represents the strict resolution rate between the step_0_, step_1_, and step_2 _trees found by the Pauprat and Rec-I-DCM3 heuristics.

Next, consider the heatmaps in Figure 2 which reflect strict consensus resolution rates for a collection of *t *trees. The heatmaps in this figure are read similarly to those in Figure 1. The difference is the interpretation of the cell (*i*, *j*). For example, on the 60 taxa dataset, cell (1, 1) represents the strict consensus resolution of the 1,508 step_0 _trees from Pauprat. Cell (2, 3) is the strict resolution rate of the step_0 _Rec-I-DCM3 trees with step_2 _Pauprat trees. High resolution rates reflect high similarity among the trees of interest. Overall, the consensus resolution rate is the highest for the step_0 _trees. This corroborates the results shown in Figure 1 that step_0 _trees are more topologically similar to each other than higher scoring trees. Furthermore, the strict resolution rate is greater among Pauprat trees than its Rec-I-DCM3 counterparts. For both Pauprat and Rec-I-DCM3, the majority resolution of comparing the top trees always resulted in a rate greater than 90% (see Figure 3). There is little variation among the step_*i *_trees when computing the majority tree. In fact, the results indicate that all of the top-scoring trees could be used to create the majority consensus tree with minimal impact on the consensus resolution rate. While it could be argued that the heatmaps in Figure 1 and Figure 2 essentially agree, Figure 1 gives greater insight into the differences found by individual trees. The consensus heatmaps, in contrast, *summarize *the information, and give a "bird's eye" view of bipartition sharing between trees found between the two approaches. By evaluating both types of heatmaps, the reader can fully appreciate the intricate similarities and differences of the two heuristics at hand, while still keeping sight of the overall picture.

### Comparisons over time

Next, we focus on the performance of Pauprat and Rec-I-DCM3 in terms of time using all of the trees returned by each search heuristic. The purpose of this experiment is to determine the information content of parsimony scores and RF rates among the set of trees found by the heuristics. Previous figures have shown that trees with the same parsimony score are distinct topologically. Now, we broaden our analysis to incorporate all trees found by a heuristic over a defined time period. Here, time is measured by the number of iterations (which is CPU time independent) and not on wall-clock time (e.g., number of hours required). Although number of iterations is an architecture-independent measure, it may not be completely adequate as each algorithm may do more work than the other per iteration. We are comparing heuristics based on their input/output behavior, which is the collection of trees returned after 5 runs of a heuristic, where each run consists of 1,000 iterations. Thus, we believe that using iterations as a basis of time is adequate for the purposes of this paper.

**Figure 4:**
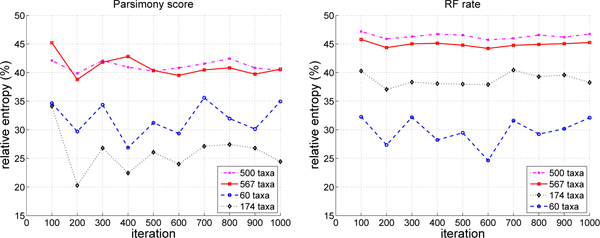
**Relative entropy for parsimony scores and RF rates for Pauprat**. Relative entropy values are computed every 100 iterations. In the plots, a data point at iteration *i *represents the average relative entropy of parsimony scores (or RF rates) based on the 100 trees produced by the Pauprat between iteration (*i *- 99) and iteration *i*.

**Figure 5:**
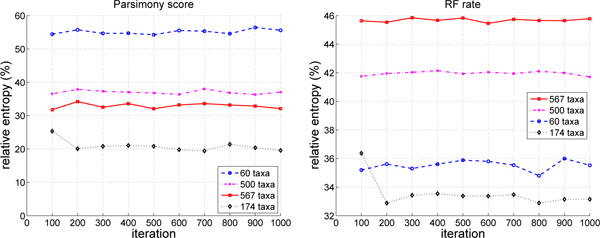
**Relative entropy for parsimony scores and RF rates for Rec-I-DCM3**. Relative entropy values are computed every 100 iterations. In the plots, a data point at iteration *i *represents the average relative entropy of parsimony scores (or RF rates) based on the 100 trees produced by Rec-I-DCM3 between iteration (*i *- 99) and iteration *i*.

Figures 4 and 5 use relative entropy as a measure for uniformly quantifying the information content of parsimony scores and RF rates. Relative entropy is shown as a percentage of the maximum possible entropy. Higher relative entropy means that there is more diversity (heterogeneity) among the values of interest, and hence higher information content. Lower relative entropy values denote homogeneous values and lower information content. One implication of low entropy values is that the search has reached a local optimum. Higher entropy values signify that more diverse trees are found by a phylogenetic heuristic, which lessen its probability of being trapped in local optima.

In Figure 4, Pauprat has a higher relative entropy than Rec-I-DCM3 when comparing parsimony scores and RF distances for the 174 and 500 taxa datasets. That is, Pauprat trees have a more diverse range of parsimony scores and RF values than Rec-I-DCM3 trees. For the 60 taxa curves, Rec-I-DCM3 has a much higher relative entropy than Pauprat. Moreover, for Rec-I-DCM3, parsimony score entropy values are much higher than RF rate values for 60 taxa. Such a result implies that the parsimony scores of trees are more diverse than their topologies. In other words, trees with different scores when compared topologically are similar. For Pauprat, the relative entropy values vary more than for Rec-I-DCM3, which has relative entropy values that are fairly constant across iterations. Essentially, such behavior denotes that the Rec-I-DCM3 search has converged as there is not much change in the parsimony or RF rates among the trees found.

## Conclusion

In this paper, we used novel methods to assess the quality of two maximum parsimony heuristics, Pauprat and Rec-I-DCM3. The goal of this work was to both ascertain the value of slower heuristics and determine if parsimony score is effective in distinguishing between different tree topologies. We designed a new entropy-based measure, which we used in tandem with parsimony scores and Robinson-Foulds (RF) distance, to quantify levels of tree heterogeneity across the Pauprat and Rec-I-DCM3 heuristics over several datasets. In addition, we used heatmaps to visualize levels of tree diversity found by the heuristics.

Our results show that parsimony score masks tree diversity in large populations of equally parsimonious trees. By using relative entropy, there is more information content in topological distance measures (such as the RF distance) than in parsimony scores. Furthermore, when considering three groups of top-scoring (step_0_, step_1_, and step_2_) trees, there is a wide-range of topological differences among these trees. In some cases, by using our relative entropy measure, parsimony scores are more diverse than the tree topologies found. This suggests that our topology-based methods may be more reliable in quantifying fine-grain differences between different heuristics, especially in larger datasets.

Our experiments with the two heuristics, show that Pauprat searches through tree space more slowly than Rec-I-DCM3. However, both Pauprat and Rec-I-DCM3 find trees with the best score. Pauprat's trees tended to be more diverse, especially on larger datasets. Pauprat's diverse collection of trees suggests its ability to escape local optima. Given that both heuristics find different classes of equally parsimonious trees, they are both useful in reconstructing phylogenies. Typical performance studies based on convergence speed of phylogenetic heuristics to the best score would discount heuristics that had a slower convergence rate. While speed is certainly important, ultimately the goal of phylogenetics is to obtain an accurate evolutionary tree for a group of taxa. Given that accuracy is typically based on a score given to a tree, we would like to be able to obtain all best-scoring trees. For the datasets studied here, our results show that both Pauprat and Rec-I-DCM3 are necessary to obtain a diverse collection of best-scoring trees. Depending on the value of these different hypotheses, our study shows that it may be worthy to improve an implementation of slower algorithms if they search a different aspect of tree space than their counterparts. In the future, we plan to create additional metrics for ascertaining levels of diversity in populations of trees. This work will also be extended to include maximum likelihood heuristics. Finally, we plan to use our results to develop new heuristics that improve the accuracy of reconstructing evolutionary trees.

## Methods

### Maximum parsimony heuristics

We study heuristics that use the maximum parsimony (MP) optimization criterion for inferring the evolutionary history between a collection of taxa. Each of the taxa in the input is represented by a molecular sequence such as DNA or RNA. These sequences are put into a multiple alignment, so that they all have the same length. Maximum parsimony then seeks a tree, along with inferred ancestral sequences, so as to minimize the total number of evolutionary events by counting only point mutations.

#### Parsimony ratchet

Parsimony ratchet is a particular kind of phylogenetic search performed with alternating cycles of reweighting and Tree Bisection Recombination (TBR). The approach works as follows: starting with an initial tree, a few of the characters (between 5 – 25%) are sampled, and reweighted. It suffices to say here that reweighting of characters involves duplicating the characters so that each shows up twice (or more) in the resulting dataset. Then, using these reweighted characters, TBR search is performed until a new starting tree is reached using this subset of data. This new starting tree is then used with the original data set to repeat the phylogenetic search. Parsimony ratchet tries to refine the search by generating a tree from a small subset of the data and using it as a new starting point. If the new tree is better than the old one, then the new one is used as the new starting tree. Otherwise, the old one is kept.

#### Rec-I-DCM3

Recursive-Iteration DCM3 (Rec-I-DCM3) [4] implements a disk-covering method (DCM) [9-11] to improve the score of the trees it finds. A DCM is a divide-and-conquer technique that consists of four stages: divide, solve, merge, and refine. At a high level, these stages follow directly from DCM being a divide-and-conquer technique.

Rec-I-DCM3, involves all of the above DCM stages, but in addition, is both recursive and iterative. The recursive part concerns the divide stage of the DCM, where overlapping subsets of the input tree's leaf nodes may be further divided into yet smaller subsets (or subproblems). This is an important enhancement to the DCM approach since for very large datasets, the subproblems remain too large for an immediate solution. Thanks to the recursion, the subproblems are eventually small enough to be solved directly using some chosen base method. At this point, Rec-I-DCM3 uses strict consensus merger to do the work of recombining the overlapping subtrees to form a single tree solution. The iterative part of Rec-I-DCM3 refers to the repetition of the entire process just described. That is, the resulting tree solution becomes the input tree for a subsequent iteration of Rec-I-DCM3.

### Comparing collections of trees

#### RF distance matrix

Given a collection of *t *evolutionary trees, we would like to quantify the topological differences that exist between them. We compute the *t *× *t *Robinson-Foulds (RF) matrix, which represents the dissimilarity between each pair of trees. Cell (*i*, *j*) in the *t *× *t *RF matrix represents the RF distance between the two trees labeled *T*_*i *_and *T*_*j*_. The Robinson-Foulds (RF) distance computes the number of bipartitions (or evolutionary relationships) that differ between them. A bipartition is an internal edge *e *of a phylogenetic tree that separates the taxa on one side from the taxa on the other. The division of the taxa into two subsets is the bipartition *B*_*i *_associated with edge *e*_*i*_. Let Σ (*T*) be the set of bipartitions defined by all edges in tree *T *. The RF distance between trees *T*_1 _and *T*_2 _is defined as

$$d_{RF}(T_{1},T_{2})=\frac{\left|\Sigma (T_{1})-\Sigma (T_{2})|+|\Sigma (T_{2})-\Sigma (T_{1})\right|}{2}.$$

Our figures plot the *RF rate*, which is obtained by normalizing the RF distance by the number of internal edges and multiplying by 100. Assuming *n *is the number of taxa, there are *n *- 3 internal edges in a binary tree. Hence the maximum RF distance between two trees is *n *- 3, which results in an RF rate of 100%. The RF rate allows us to compare topological differences when the number of taxa is different. Thus, the RF rate varies between 0% and 100% signify that trees *T*_1 _and *T*_2 _are identical and maximally different, respectively.

#### Relative entropy

Entropy represents the amount of chaos in the system. We use entropy to quantitatively capture the distribution of parsimony scores and RF rates among the collection of trees of interest. In our plots, we show *relative entropy*, which is a normalization of entropy, to allow the comparison of entropy values across different population sizes. Relative entropy ranges from 0% to 100%. Higher entropy values indicated more diversity (heterogeneity) among the population of trees. Lower entropy values indicate less diversity (homogeneity) in the population.

Let *λ *represent the total number of objects (parsimony scores or RF rates) in the population of trees. For example, suppose we want to partition a population of 10 trees based on their parsimony scores. Then, *λ *= 10. However, if we are interested in partitioning the 10 trees based on the upper triangle of the corresponding 10 × 10 RF matrix, then $\lambda =\frac{10(9)}{2}$ or 45 since the RF matrix is symmetric. Next, we group the *λ *objects into *P *total partitions. Each partition *i *contains *n*_*i *_individuals with identical values. For RF, each individual in partition *i *will have the same RF value. An individual in the RF matrix refers to a cell location (*p*, *q*).

We can compute the entropy (*E*_*T*_) of the collection of parsimony scores as:

$$E_{T}=-\sum_{i}^{P}p_{i}\log p_{i},$$

where *p*_*i *_= $\frac{n_{i}}{\lambda }$. The highest entropy value (*E*_*max*_) is log *λ *. Relative entropy (*E*_*rel*_) is defined as the quotient between the entropy *E*_*T *_and the maximum entropy *E*_*max *_and multiplying by 100 to obtain a percentage. Thus,

$$E_{rel}=\frac{E_{T}}{E_{max}}\times 100.$$

#### Resolution rate

For *n *taxa, a resolved, unrooted binary tree will have *n *- 3 bipartitions (or internal edges). Trees with less than *n *- 3 bipartitions are considered to have unresolved relationships among the *n *taxa. In general, binary (or 100% resolved) trees are preferred by life scientists. The *resolution rate *of a tree is the percentage of bipartitions that are resolved. One common use of this measure is related to evaluating consensus trees, which are used to summarize the information from a set of *t *trees. The strict consensus method returns a tree such that the bipartitions of the tree are only those bipartitions that occur in all of the *t *trees. The majority consensus tree incorporates those bipartitions that occur in at least 50% of the *t *trees of interest. Highly resolved consensus trees denote that a high degree of similarity was found among the collection of trees.

### Experimental methodology

#### Datasets

We used the following biological datasets as input to study the behavior of the maximum parsimony heuristics.

1. A 60 taxa dataset (2,000 sites) of ensign wasps composed of three genes (28S ribosomal RNA (rRNA), 16S rRNA, and cytochrome oxidase I (COI)) [12]. The best-known parsimony score is 8,698, which was established by both Pauprat and Rec-I-DCM3.

2. A 174 taxa dataset (1,867 sites) of insects and their close relatives for the nuclear small subunit ribosomal RNA (SSU rRNA) gene (18S). The sequences were manually aligned according to the secondary structure of the molecule [13]. The best-known parsimony score is 7,440, which was established by both Pauprat and Rec-I-DCM3.

3. A set of 500 aligned *rbcL *DNA sequences (759 parsimony-informative sites) [14] of seed plants. The best-known parsimony is 16,218, which both Pauprat and Rec-I-DCM3 found.

4. A set of 567 "three-gene" (*rbcL*, *atpB*, and *18s*) aligned DNA sequences (2,153 sites) of angiosperms [15]. The best-known parsimony score is 44,165, which both Pauprat and Rec-I-DCM3 found.

#### Starting trees

All methods used PAUP*'s random sequence addition module to generate the starting trees. First, the ordering of the sequences in the dataset is randomized. Afterwards, the first three taxa are used to create an unrooted binary tree, *T *. The fourth taxon is added to the internal edge of *T *that results in the best MP score. This process continues until all taxa are added to the tree. The resulting tree is then used as the starting tree for a phylogenetic analysis.

#### Implementation and platform

We set the parameters of the Pauprat and Rec-I-DCM3 algorithms according to the recommended settings in the literature. We use PAUP* [6] to analyze our four datasets using the parsimony ratchet heuristic. The implementation of the parsimony ratchet was implemented using PAUP* [6]. For our analysis, we randomly selected 25% of the sites and doubled their weight; initially, all sites are equally weighted. On each dataset, we ran 5 independent runs of the parsimony ratchet, each time running the heuristic for 1,000 iterations. For Rec-I-DCM3, it is recommended that the maximum subproblem size is 50% of the number of sequences for datasets with 1,000 or less sequences and 25% of then number of sequences for larger datasets not containing over 10,000 sequences. We used the recommended settings established by Roshan et. al [4] for using TNT as a base method within the Rec-I-DCM3 algorithm.

We used the HashRF algorithm [16,17] to compute the RF distances between trees. Each heuristic was run five times on each of the biological datasets. All experiments were run on a Linux Beowulf cluster, which consists of four, 64-bit, quad-core processor nodes (16 total CPUs with gigabit-switched interconnects). Each node contains four, 2 GHz AMD Opteron processors and they share 4 GB of memory. We note that both Rec-I-DCM3 and parsimony ratchet are sequential algorithms. The parallel computing environment was used as a way to execute multiple, independent batch runs concurrently.

## Competing interests

The authors declare that they have no competing interests.

## Authors' contributions

TW ran the maximum parsimony algorithms to obtain the phylogenetic trees studied in this manuscript. SS and SM wrote the algorithms to analyze the data from the maximum parsimony experiments. SS created all of the plots. All authors contributed to writing the manuscript and have approved its final contents.
